# The impact of social isolation and loneliness on cardiovascular disease risk factors: a systematic review, meta-analysis, and bibliometric investigation

**DOI:** 10.1038/s41598-024-63528-4

**Published:** 2024-06-04

**Authors:** Osama albasheer, Siddig Ibrahim Abdelwahab, Mohammad R. Zaino, Ahmed Abdallah Ahmed Altraifi, Nasser Hakami, Ehab I. El-Amin, Mohammed M. Alshehri, Saeed M. Alghamdi, Abdulfattah S. Alqahtani, Aqeel M. Alenazi, Bader Alqahtani, Ahmed Alhowimel, Shadab Uddin, Husam Eldin Elsawi Khalafalla, Isameldin E. Medani

**Affiliations:** 1 Family and Community Medicine Department, Faculty of Medicine, College of Medicine, Jazan University, 45142 Jazan, Saudi Arabia; 2 Health Research Centre, Jazan University, Jazan, Saudi Arabia; 3 Physical Therapy Department, Faculty of Applied Medical Sciences, Jazan University, Jazan, Saudi Arabia; 4 Obstetrics and Gynaecology Department, College of Medicine, Jazan University, Jazan, Saudi Arabia; 5 Surgical Department, College of Medicine, Jazan University, Jazan, Saudi Arabia; 6 Department of Epidemiology, College of Public Health and Tropical Medicine, Jazan University, Jazan, Saudi Arabia; 7 Department of Physical Therapy, Faculty of Applied Medical Sciences, Jazan University, Jazan, Saudi Arabia; 8 Clinical Technology Department, Respiratory Care Program, Faculty of Applied Medical Sciences, Umm Al-Qura University, Makkah, Saudi Arabia; 9 Department of Rehabilitation Sciences, College of Applied Medical Sciences, King Saud University, Riyadh, Saudi Arabia; 10 Health and Rehabilitation Sciences, Prince Sattam Bin Abdulaziz University, Al-Kharj, Saudi Arabia; 11 Department of Health Education and Promotion, School of Nutrition and Translational Research in Metabolism, Maastricht University, Maastricht, The Netherlands

**Keywords:** Social health, Loneliness, Social isolation, Cardiovascular disease, Meta-analysis, Bibliometrics, Neuroscience, Cardiology, Diseases, Medical research, Risk factors

## Abstract

Data on the association between social isolation, loneliness, and risk of incident coronary heart disease (CVD) are conflicting. The objective of this study is to determine the relationship between social isolation and loneliness, and the risk of developing cardiovascular disease (CVD) in middle age and elderly using meta-analysis. The purpose of the bibliometric analysis is to systematically evaluate the existing literature on the relationship between social isolation, loneliness, and the risk of developing cardiovascular disease (CVD) in middle-aged and elderly individuals. A comprehensive search through four electronic databases (MEDLINE, Google Scholar, Scopus, and Web of Science) was conducted for published articles that determined the association between social isolation and/or loneliness and the risk of developing coronary heart disease from June 2015 to May 2023. Two independent reviewers reviewed the titles and abstracts of the records. We followed the Preferred Reporting Items for Systematic Reviews and Meta-Analyses guideline to conduct the systematic review and meta-analysis. Data for the bibliometric analysis was obtained from the Scopus database and analyzed using VOSviewer and Bibliometrix applications. Six studies involving 104,511 patients were included in the final qualitative review and meta-analysis after screening the records. The prevalence of loneliness ranged from 5 to 65.3%, and social isolation ranged from 2 to 56.5%. A total of 5073 cardiovascular events were recorded after follow-up, ranging between 4 and 13 years. Poor social relationships were associated with a 16% increase in the risk of incident CVD (Hazard Ratio of new CVD when comparing high versus low loneliness or social isolation was 1.16 (95% Confidence Interval (CI) 1.10–1.22). The bibliometric analysis shows a rapidly growing field (9.77% annual growth) with common collaboration (6.37 co-authors/document, 26.53% international). The US leads research output, followed by the UK and Australia. Top institutions include University College London, Inserm, and the University of Glasgow. Research focuses on "elderly," "cardiovascular disease," and "psychosocial stress," with recent trends in "mental health," "social determinants," and "COVID-19". Social isolation and loneliness increase the risk of and worsen outcomes in incident cardiovascular diseases. However, the observed effect estimate is small, and this may be attributable to residual confounding from incomplete measurement of potentially confounding or mediating factors. The results of the bibliometric analysis highlight the multidimensional nature of CVD research, covering factors such as social, psychological, and environmental determinants, as well as their interplay with various demographic and health-related variables.

## Introduction

Many people around the world experience social isolation and loneliness. The National Academies of Sciences, Engineering, and Medicine (NASEM) published a report in 2020 on social isolation and loneliness that indicated that nearly one-fourth (24%) of Americans aged 65 and older were socially isolated^1^. The World Health Organization (WHO) has noted that social isolation and loneliness among older adults are growing health, social, and financial concerns^2^. Social isolation is defined as having infrequent social contact or lacking social integration. Loneliness is defined as 'perceived isolation or loss of companionship that is distressing to the individual^1,3^. The terms 'social isolation' and 'loneliness' are often used interchangeably in research^4^.

Adults who are socially isolated and lonely are more likely to die young^5–8^. Because of changes in their physical health (chronic illnesses, vision, hearing, memory loss, disability, and loss of mobility) and social connections (loss of family and friends and living alone), older adults are more likely to experience social isolation and loneliness^9,10^.

There is mixed evidence regarding the relationship between social isolation, loneliness, and the risk of developing Cardiovascular Disease (CVD)^11^. A meta-analysis of 19 studies found that social isolation or loneliness was associated with an increased risk of incident CVD (pooled relative risk [RR], 1.29 [95% CI, 1.04–1.59]^12^. However, data on the association between social isolation, loneliness, and risk of incident CVD is inconsistent. Since then, substantial research has examined the complex and intertwined relationship between social health and incident CVD. One UK study found that, in unadjusted analyses, social isolation and loneliness were associated with the incidence of acute Myocardial Infarction (MI) in people without CVD at the start of the study^13^. Smith et al. discovered that social isolation only has a direct impact on incident CVD in an analysis of two extensive prospective studies^14^. The English Longitudinal Study of Aging's prospective data also came to the same conclusion: there was no evidence of a long-term cumulative effect of social relationships on the risk of cardiovascular disease^15^. More recently, a narrative review suggested that social health and incident CVD have a bi-directional relationship and share risk factors^16^. The main objective of this study is to determine the relationship between social isolation and loneliness, and the risk of developing cardiovascular disease (CVD) in middle age and elderly (age ≥ 35 years). These age groups are more vulnerable to loneliness and social isolation and this can have a serious impact on their health. It was reported that more than one-third of adults aged 45 and older are considered lonely, and about one-fourth of adults aged 65 and older are considered to be socially isolated^1^. Furthermore, this study aimed to investigate the impact of social relationships on the incidence of cardiovascular disease and try to answer the question: does the association between social relationships and disease incidence vary depending on the dimension of relationships measured and individual and contextual factors? The findings of the manuscript will help to inform our understanding of the complex relationship between social health and CVD in older adults. This information can be used to develop interventions to reduce social isolation and loneliness, and ultimately to improve the cardiovascular health of older adults. This study's objective is extended to thoroughly examine the existing literature, statistical synthesis, and bibliometric analysis to understand better the interconnections between social isolation, loneliness, and risk factors for cardiovascular disease. This includes analyzing publication patterns, citation networks, and research trends to assess the quantity and impact of research in this field. The study aims to identify key contributors and influential studies, providing valuable insights into the relationship between social factors and cardiovascular health.

## Methods

The Preferred Reporting Items for Systematic Reviews and Meta-Analyses (PRISMA) guideline was used^17^ to conduct the systematic review, bibliometric study and meta-analysis presented here, and our procedure followed it unless stated otherwise.

**Figure 1:**
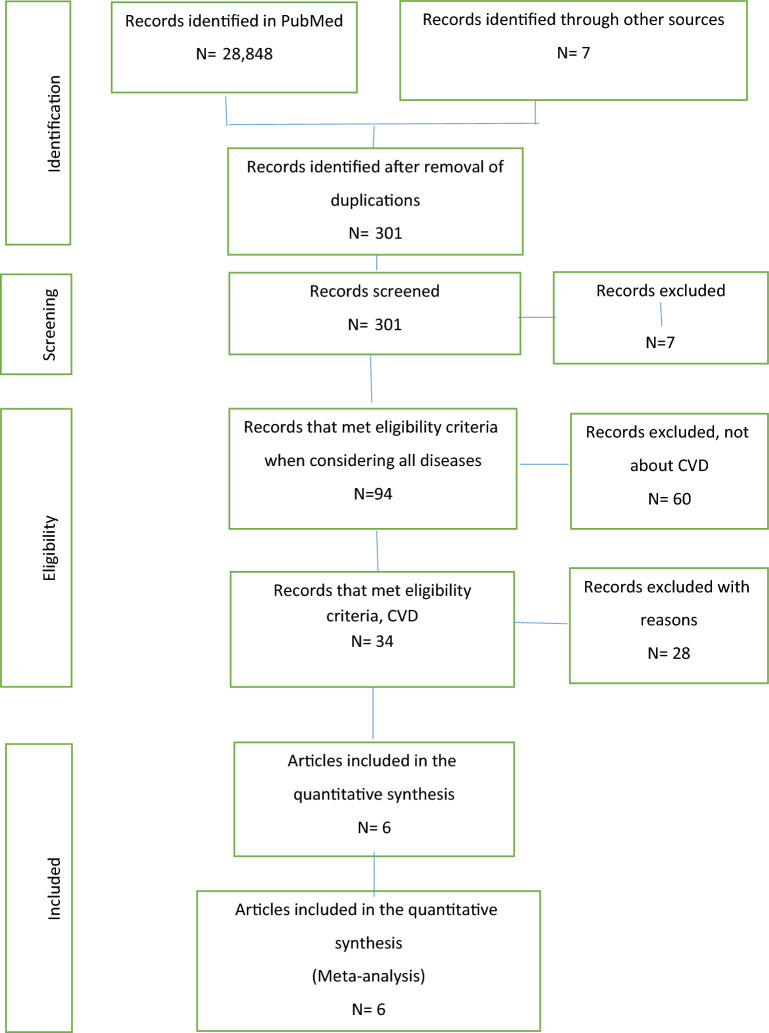
The flowchart of the process of meta-analysis.

The flowchart of the process of this meta-analysis is displayed in Fig. 1. Our study identified a total of 28,855 articles that had relevant information for this analysis. By reviewing titles and abstracts, we excluded 28,554 for different reasons, including duplicate records, review articles, case reports, nonclinical studies, and participants not meeting the inclusion criteria. Then, we screened 103 full-text articles for further assessment and excluded 97. Finally, we included six studies with a total of 104,511 participants.

### Ethical approval

Our study did not require an ethical board approval because systematic reviewers do not collect deeply personal, sensitive or confidential information from participants.

### Consent for participation

Consent for participation already obtained by the authors of the selected studies.

## Meta-analysis and systematic review

### Eligibility criteria

#### Studies were eligible for inclusion if they met the following criteria

(a) New incident of cardiovascular disease (CVD) among individuals due to loneliness and/or social isolation and/or social support between December 2015 and December 2022. (b) CVD defined as diagnoses listed under codes I20–I25 of the 10th revision of the International Statistical Classification of Diseases and Related Health Problems (ICD-10). (c) If a person has a subjectively unpleasant feeling connected to the belief that their interactions with others are inadequate, they are deemed lonely. Metrics of social isolation align with an objective metric that quantifies the lack of connections, affiliations, or interpersonal interactions. (d) Longitudinal studies with reported adjusted hazard ratios for CVD.

### Exclusion criteria

#### Studies were excluded if they met the following criteria:

(a) Incident CVD was not the first time that participants had been diagnosed. (b) They were certain publication types (e.g., letters, case reports, meta-analysis, comments). (c) They had insufficient or duplicate data. Insufficient data refers to missing information such as incomplete demographic information or insufficient in term of sample size and events. Duplicate data refers to multiple instances of the same or very similar information within a dataset or database leading to unnecessary redundancy. (d) They were non-English studies.

### Literature search strategy

A comprehensive literature search was conducted in four electronic databases (MEDLINE, Google Scholar, Scopus, and Web of Science) for articles that evaluated the association between social isolation and/or loneliness and the risk of developing CVD from June 2015 to May 2023. An English language restrictions were applied. Free text terms (loneliness, social isolation, social relationships, social support, and social network) were searched first and then combined with the disease outcome. Duplicates were removed, and then two researchers independently screened titles and abstracts before assessing full records using a standardized screening sheet. In addition to the electronic database searches, we also conducted citation chaining using relevant journals, reference lists, and conference abstracts. We manually searched the reference lists of all included studies to identify potentially relevant studies not captured by the electronic database search. The relevant journals were searched to ensure that studies published in these journals are not missed during the review process.

By examining the reference lists, we wanted to find relevant studies that were not initially identified through the search strategy. In addition, the conference abstracts can provide unpublished research findings that are not yet published as full papers in journals. This can help capture the most up-to-date research on the topic.

### Data extraction

Two researchers independently screened the titles and abstracts of the retrieved studies. If the necessary information was not available in the titles and abstracts of the studies, the full text was reviewed. The details extracted for each study were first author, data source and country, year of publication, participants (sample size, sex, and age), outcome measures, number of events, follow-up duration in years, main results, terms used for social network, category of cardiovascular disease, measurement tools, and maximum adjusted covariates.

### Quality assessment

The quality of all included articles was assessed using the Agency for Healthcare Research and Quality framework designed for observational studies^18^. The validated items included the following: sampling bias, missing data, non-response bias, differential attrition, information error with regard to exposure and outcome measure, detection bias, confounding, and study size. We considered socioeconomic status, gender, and age as potential confounders^19,20^. No article was excluded based on its overall quality rating; instead, subgroup and sensitivity analyses were performed to assess whether the findings were robust to differences in internal validity^21^.

### Data collection and analysis

To answer the questions related to the objectives of this study, we conducted a preliminary synthesis by grouping study characteristics and results according to their measure of relationships. We assessed consistency and heterogeneity between the articles using Cochran’s Q test at a significance level of *p* < 0.10^22^. We defined a statistic I^2^ of 50% or more as a substantial level of heterogeneity^23^. This meta-analysis used hazard ratios (HRs) with confidence intervals (CIs) as common risk estimates. Most of the studies in this review reported relative hazards of new diagnoses. We excluded articles that reported odds ratios (ORs) or other measures different from HRs.

We used a random effects model because it provides more conservative results than a fixed effects model^24^. Random effects models provide a more conservative estimate of the overall effect size, taking into account the potential heterogeneity across studies. Using random effect allow us to generalization of the findings to a broader set of studies beyond those included in the review. Furthermore, our research question concerned with estimating an average effect across a diverse range of studies. We assumed that the samples from the populations were distinct (independent samples). We calculated the overall effect size and 95% CI using the inverse variance weighted method. The included studies used the terms "social isolation" and/or "loneliness." If several studies reported results from the same cohort, we selected the findings with the longest follow-up time. If several HRs were presented in one study, we extracted the most completely adjusted HR. We performed a random effects model weighted by the inverse variance method to pool the logarithm of the hazard ratio (logHR) and the Standard Error Provided Hazard Ratio (SElogHR). "logHR" is a measure often used in estimating the HR of an event occurring in different groups. On the other hand, "SElogHR" quantifies the uncertainty or variability associated with the estimation of the logarithm of the hazard ratio and is crucial for determining the statistical significance of the effect size. Moreover, we performed subgroup and sensitivity analyses when appropriate to test whether internal study validity and small-study effects affected our overall results. We used a funnel plot to assess the degree of possible publication bias. A visually significant asymmetry in the funnel plots indicated major publication bias. All the statistical analyses were performed using Review Manager Version 5.30 software (the Cochrane Collaboration, Copenhagen, Denmark)^25^. We created the graphical charts using the functions available in the Metafor package in R software (Version 4.1.0 for Windows).

## Bibliometric study

### Database selection and search strategy

Scopus was chosen for searching the bibliographic data due to its comprehensive coverage, advanced search functionalities, and citation data, enabling targeted searches and robust bibliometric analysis^26^. The terms for social isolation, loneliness, and cardiovascular disease risk were obtained from the MeSH database^27^, including "Social Isolation," "Loneliness," "Ostracism," "Social Alienation," and "Social Deprivation." To conduct the search in the Scopus database, a query was constructed using these terms and Boolean operators. The search aimed to find articles that mentioned these concepts in relation to "Cardiovascular Diseases," "Adverse Cardiac Event," "Cardiac Events," or "Major Adverse Cardiac Events." The initial search yielded 1,090 document results.

### Inclusion criteria

The inclusion criteria for the study were refined to focus on data-driven studies and ensure relevance. Specifically, articles were included if they met the following criteria: (1) document type: "ar" (articles), (2) publication stage: "final," and (3) language: English. This refinement resulted in the identification of 628 relevant documents. The data extracted from these documents were saved in CSV and BibTeX formats for further analysis.

### Bibliometric analysis

#### Tools

The bibliometric analysis of this data was conducted using VOSviewer (Version 1.6.20) and Bibliometrix (Version 4.2.2) applications^28,29^. Bibliometric analysis techniques include visualizing and analyzing bibliometric networks to explore relationships between authors, institutions, and keywords based on co-occurrence or citation patterns. Advanced algorithms generate visualizations like co-authorship networks and term maps, aiding in identifying related research, influential authors, and emerging trends. Bibliometric analysis tools allow calculation of bibliometric indicators such as publication and citation counts, co-authorship statistics, and generate graphical representations like bar charts and network graphs. These techniques provide insights into productivity, impact, and collaboration patterns within a research field^28,30,31^.

### Performance analysis

The average age of the documents represents the average time since their publication and can be calculated by subtracting the publication year from the current year. This provides insights into the age distribution of the included studies. The calculation involves summing the ages of all documents and dividing it by the total number of documents. Additionally, international collaborations are identified by examining the affiliations of authors listed in the publications. Instances where authors from different countries collaborate on a research project are considered as international collaborations.

### Thematic map and evolution and trending topics

The Bibliometrix (Version 4.2.2) software generated the thematic map, which was divided into four quadrants representing different research areas. The themes within these quadrants were classified based on their centrality (relevance degree) and density (development degree). Centrality measured the relevance of a theme within the research network by assessing its connections to other themes. At the same time, density represented the level of interconnections and collaborations among researchers in a given theme. The four quadrants consisted of niche themes characterized by low centrality and density, basic themes with low centrality but high density, motor themes displaying high centrality and density, and decline or emerging themes exhibiting high centrality but low density. This approach allowed for a visual representation of the research areas, highlighting their relative importance and development within the network. The Bibliometrix software and the centrality and density criteria were instrumental in generating the insightful thematic map. The thematic evolution analysis of the knowledge dynamicity in the field of social isolation and the risk of cardiovascular disease was conducted using the Bibliometrix software. Trending topics was analyzed using author’s keywords and the respective graph was generated using the Bibliometrix application. The graph depicts the time span of the research topic, with horizontal lines indicating the duration, and blue circles representing the frequency of the term^28,31^.

## Results

### Baseline characteristics of included studies

This study included six observational studies conducted in Australia, Sweden, the United Kingdom, Denmark, England, and Germany^8,32–36^. All trials enrolled males and females except one, which involved women from the Women's Health Initiative Extension Study II^8^. Moreover, the period of follow-up ranged between 4 to 13 years. The primary outcomes were incident CVD, AMI, and death from CVD. The characteristics of the included studies and participants are summarized in Table 1 and Appendix 1 and 2.

**Table 1:** Baseline characteristics of the included studies.

Population characteristics across included studies	
Total number of studies	6
Total number of participants	104,511
Age of participants	≥ 35
Data source & Country	Aspirin in Reducing Events in the Elderly (ASPREE) trial/Australia, Swedish national registers in Gothenburg/ Sweden, Women’s Health Initiative Extension Study II/ US, 2013 Danish “How are you?” survey/Denmark, Wave 4 participants of English Longitudinal Study of Ageing (ELSA)/ England, and Population-based Heinz Nixdorf Recall study/ Germany
Term used for social health	Loneliness in four papers^8,24–26^ Social isolation in two papers^27,28^
Study characteristic	
Data collection dates, range	2000–2017
Length of follow-up, range	4–13 years
Gender	All-Female sample in one paper^8^ Mixed sample in five papers^24–28^
Total number of recorded cardiovascular events	5073

### Assessment of social health

Four cohorts measured the prevalence of loneliness and described its association with incident CVD^8,32–34^. The prevalence of loneliness ranged from 5 to 65.3%. Two cohorts evaluated social isolation with prevalence ranging from 2 to 56.5%^35,36^. The assessment scales used to measure loneliness were as follows: Center for Epidemiological Studies – Depression (CESD) Scale^37^, the UCLA Loneliness Scale^38^, Single question loneliness scale ^39^, Three-Item Loneliness Scale [T-ILS]^40^, and three-item subscale of the revised University of California, Los Angeles loneliness scale^41^. The measurement tools used in the evaluation of social isolation were the Berkman-Syme Social Network Index (SNI)^42^ and the five-item Shankar index^43^; see Appendix 3 and 4.

### Assessment of cardiovascular diseases

Across the studied cohorts, 5073 cardiovascular events were recorded (Table 1). The main outcomes were myocardial infarction, angina pectoris, heart attack, heart failure hospitalization, and death from CVD^32,34–36^. Cases of CVD were identified from self-reported physician-diagnosed conditions and administrative records^35,36^. Information on deaths was obtained from the national mortality registry^35^.

### Study validity

**Figure 2:**
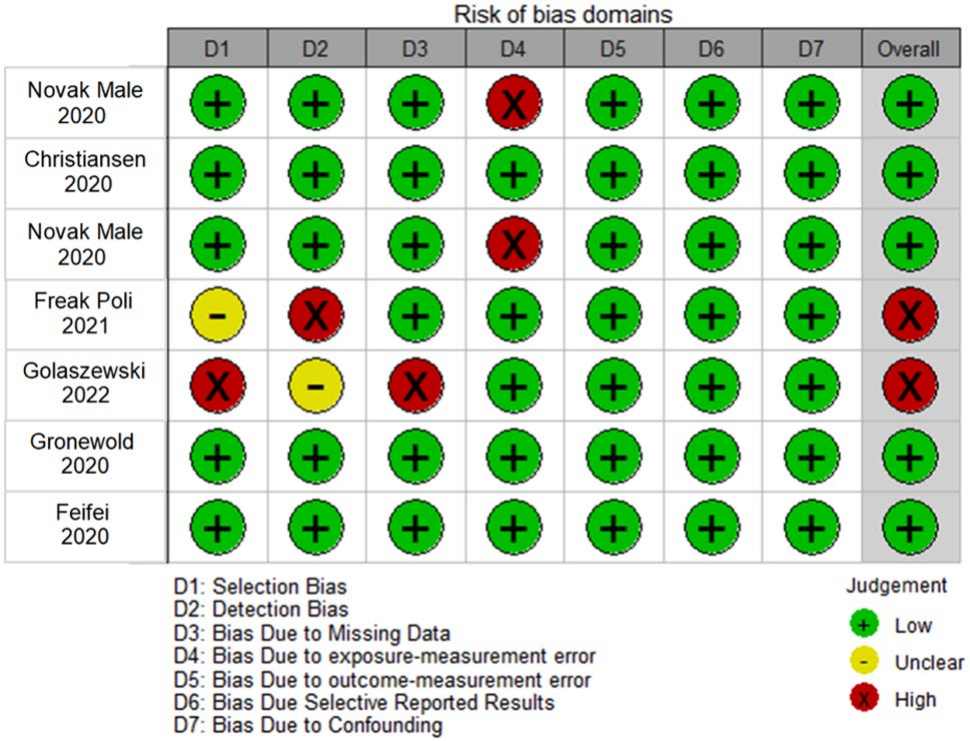
Risk of bias across the studies involved in this meta- analysis.

Figure 2 summarizes the risk of bias across the studies involved in this meta-analysis. The validity assessment of the selected cohorts was based on the following domains: “Selection bias” refers to systematic differences between the participants who are included or excluded in the study; it can introduce bias and affect the generalizability of the findings. “Detection bias” relates to biases that arise from differences in the detection or ascertainment of outcomes between the study groups. “Bias due to missing data” is a concern in longitudinal studies when participants have missing data on key variables over time. “Bias due to exposure measurement error” refers to inaccuracies or imprecisions in measuring the exposure variable over time. “Bias due to outcome measurement error” arises from inaccuracies or imprecisions in measuring the outcome variable over time. “Selective reporting bias” when results are not fully or accurately reported. “Confounding bias” domain helps in adequate control for confounding variables to minimize bias and obtain accurate estimates of the exposure-outcome relationship, see Appendix 5. Five cohorts reported an overall low risk of bias^29–31^. Two studies reported a high risk of bias due to limited information on blinding of outcome assessments and missing data^8^. In one study, the risk of bias was unclear due to limited information on blinding of outcome assessment and deviations from the intended intervention. In summary, the studies by Christiansen 2020, Gronewold 2020, and Feifei 2020 are considered to have a relatively low risk of bias across all evaluated domains. However, the other studies have some concerns or potential biases in one or more domains, such as selection, missing data, exposure measurement error, outcome measurement error, selective reporting of results, and confounding. Additional models adjusting for socio-demographic, behavioral, and psychological factors were applied in all included cohorts.

**Figure 3:**
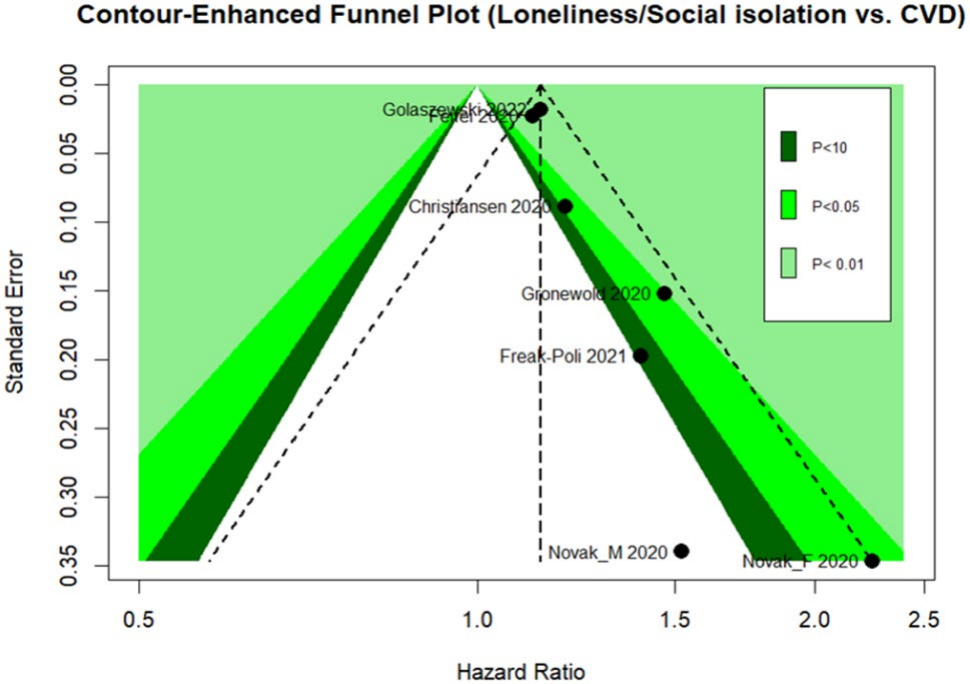
Contour-enhanced funnel plots.

Overall, the data set shows a symmetrical pattern in the funnel plot that might be indicative of publication bias. Contour-enhanced funnel plots include colors that signify the significance level of each study in the plot (Fig. 3). There is only one study^33^ with a large standard error that has no significant effect but still follows the funnel pattern of symmetry. The limited number and some kind of heterogeneity of involved studies did not support the use of tests for funnel plot asymmetry^44^.

### Social health and cardiovascular diseases

**Figure 4:**
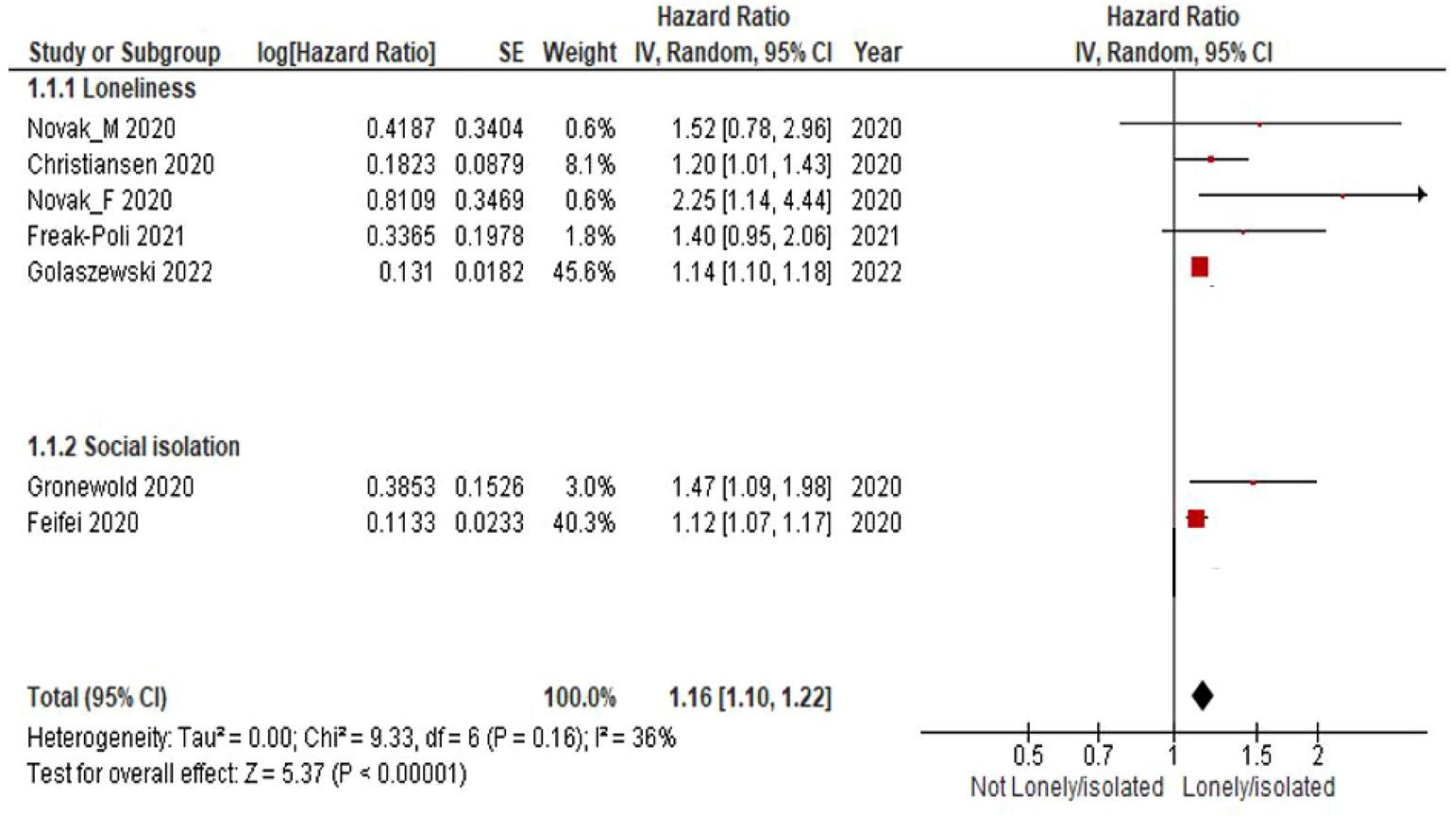
Predictors of cardiovascular and all-cause mortality in older men and women. Novak_ M = Novak_ Male, Novak_ F = Novak_ Female.

One study examined whether loneliness predicts cardiovascular and all-cause mortality in older men and women and reported that cardiovascular disease accounted for 59.2% of all deaths in the selected cohort^33^. Across the remaining studies, 5,073 CVD events were reported, and the Hazard Ratio (HR) of new CVD when comparing high versus low loneliness or social isolation was 1.16 (95% CI 1.10 to 1.22; see Fig. 4). In another words the poor social relationships were associated with a 16% increase in risk of incident CVD (*P* < 0.0001). The large *P*-value for heterogeneity of 0.16 (> 0.05) and the l^2^ of 36% both indicate that heterogeneity was not present. So we didn’t need to perform subgroup analyses according to risk of confounding and risk of bias due to outcome measurement error.

### Bibliometric analysis

#### Overview of the bibliographic data

**Figure 5:**
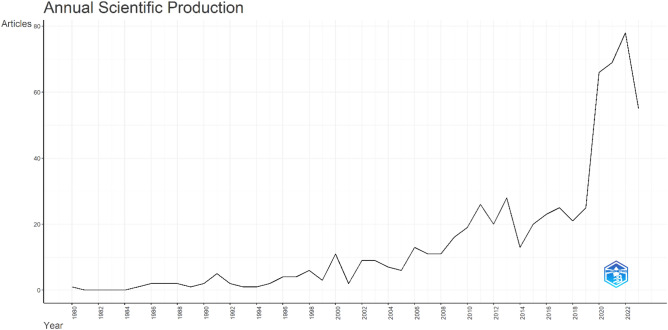
Annual production of scholarly work related to social isolation, loneliness, and cardiovascular disease risk. Y-axis: the number articles published. X-axis: the years since the first article published in the topic of this paper. This figure was generated using the Bibliometrix (version 4.2.2) application and the BibTex data file.

The analysis covers a timespan from 1980 to 2023 and includes 628 documents from 373 journals. For the meta-analysis, the timeframe from June 2015 to May 2023 was chosen to provide an up-to-date synthesis of the literature. As shown in Fig. 5, the average annual growth rate of the analyzed documents is 9.77%, indicating a growing interest in this field. The average age of the documents is 8.24 years, suggesting the inclusion of both recent and older studies. On average, each document received 52.56 citations, highlighting scholarly attention. Collaboration among authors was prevalent, with an average of 6.37 co-authors per document, and approximately 26.53% of co-authorships involved international collaborations, emphasizing the global nature of the research.

#### Hotspots

**Figure 6:**
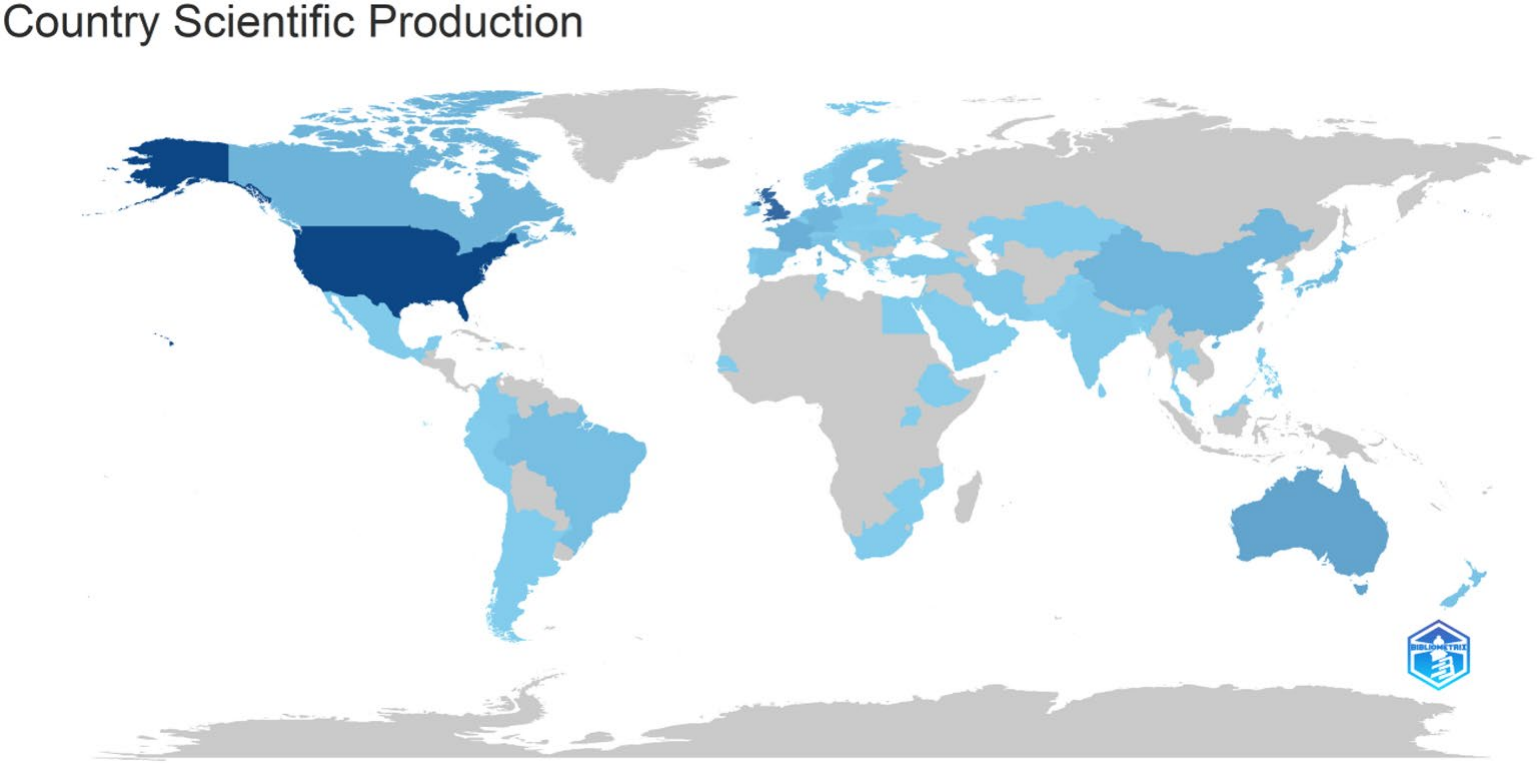
Global production of scholarly work related to social isolation, loneliness, and cardiovascular disease risk. Countries with a dark blue color are the most productive. Countries outside the blue category have not made any contributions to research in this particular area. This figure was generated using the Bibliometrix (version 4.2.2) application and the BibTex data file*.*

In the field of social isolation, loneliness, and cardiovascular disease risk, several prolific authors have made significant contributions. A total of 3485 authors contributed to the analyzed documents, with 34 authors responsible for single-authored works. Steptoe, A. affiliated with the University College London, United Kingdom, stands out with 17 publications, followed by Woodward, M. with 10 and Petersen, I. with 7. The United States has been the most prominent country in producing research, with 213 publications, followed by the United Kingdom with 162 and Australia with 53 (Fig. 6). University College London leads as the most productive institution with 41 scholarly works, alongside Inserm and the University of Glasgow, each contributing 16. Notable journals include the International Journal of Environmental Research and Public Health, Social Science and Medicine, and Psychosomatic Medicine, publishing 19, 19, and 18 works, respectively. These contributions from authors, countries, institutions, and journals have enriched our understanding of the relationship between social isolation, loneliness, and cardiovascular disease risk.

#### Most cited countries

The United States (USA) is the most cited country, with a total citation count (TC) of 9767 and an average of 81.40 citations per article. The United Kingdom follows closely with a TC of 8474 and an average of 79.90 citations per article. Australia has a lower TC of 621 but with an average of 18.80 citations per article. The Netherlands has a TC of 576 and an average of 32.00 citations per article. Canada has a TC of 575 and an average of 25.00 citations per article. Sweden has a TC of 517 and an average of 47.00 citations per article. These statistics indicate the influence and impact of research from these countries in the field, with the United States and the United Kingdom leading the way in terms of citation counts.

#### Intellectual structure

**Figure 7:**
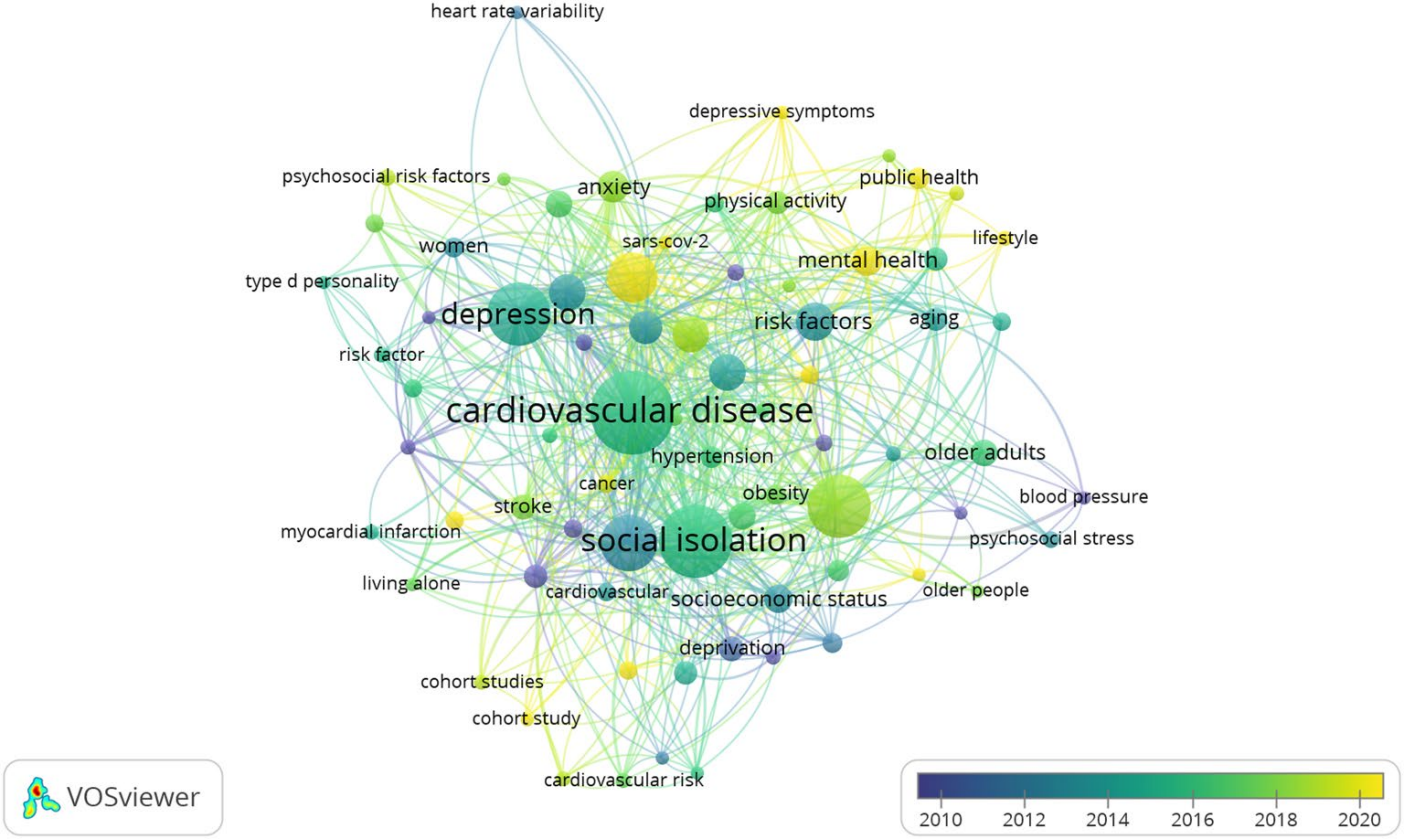
Most Frequent Keywords. This figure was generated using the VOSviewer (version 1.6.20) application and the BibTex data file.

The analysis includes 1224 author's keywords. Figure 7 presents the most frequent keywords (n = 74), offering an indicator of the intellectual structure in the field. Among the most frequent keywords, “cardiovascular disease” stands out with 90 occurrences, representing 3.74% of the total. Social isolation follows closely with 74 occurrences (3.08%), while depression and loneliness appear 58 times (2.41%) and 57 times (2.37%), respectively. Other significant keywords include mortality (48 occurrences, 2.00%), COVID-19 (39 occurrences, 1.62%), risk factors (25 occurrences, 1.04%), cardiovascular diseases (24 occurrences, 1.00%), epidemiology (24 occurrences, 1.00%), and stress (24 occurrences, 1.00%). These keywords collectively reflect the important themes and topics within the intellectual structure of the field, emphasizing the role of cardiovascular disease, social isolation, mental health factors, and their impact on mortality and public health, including the context of the COVID-19 pandemic.

#### Thematic map

**Figure 8:**
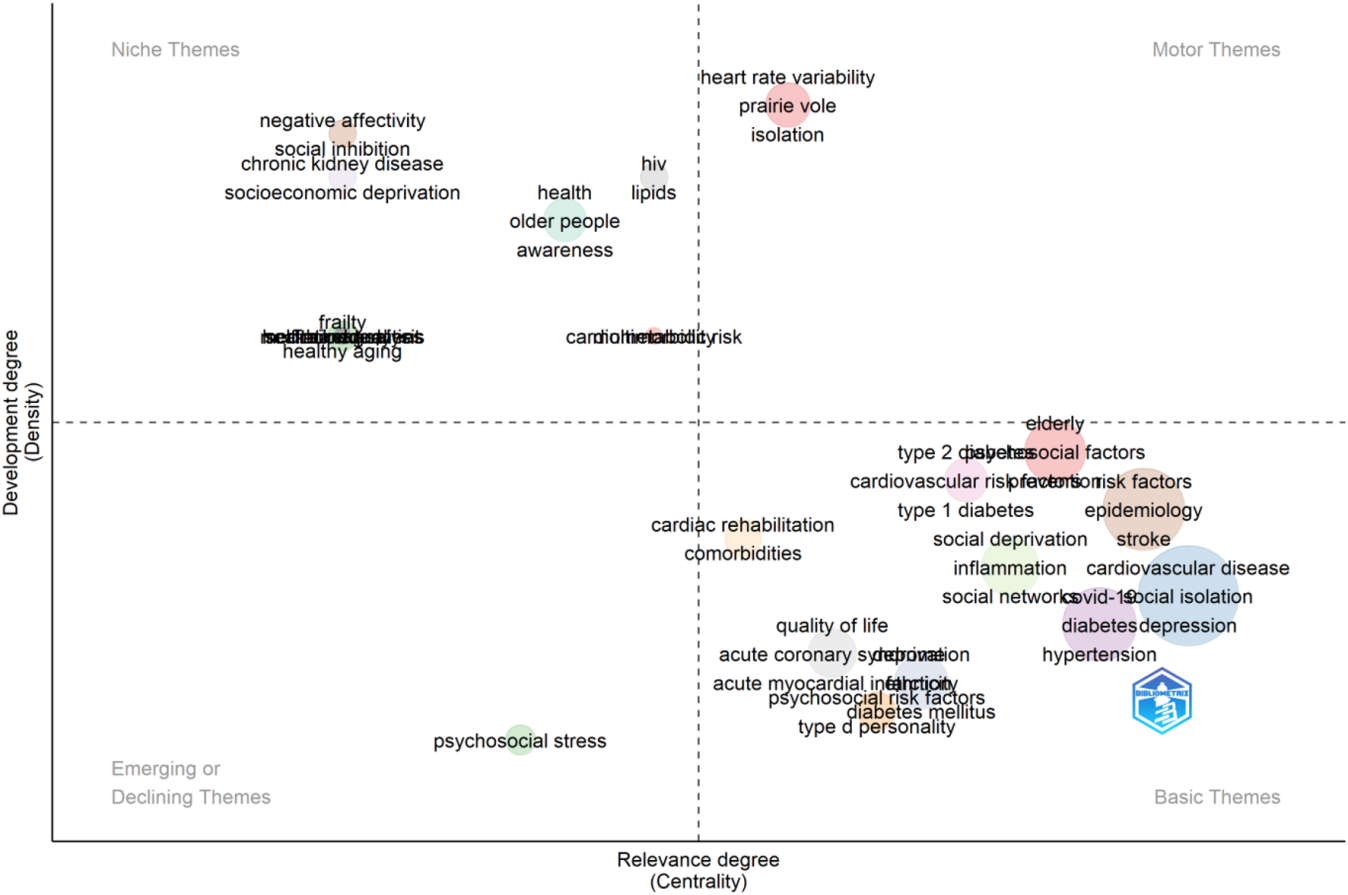
Thematic map. Thematic maps are divided into four quadrants based on centrality and density, which represent the importance and development of research topics. This figure was generated using the Bibliometrix (Version 4.2.2) application and the BibTex data file.

The clusters presented in Table 2 and Fig. 8, illustrate various research themes in the field, along with their corresponding Callon Centrality and Callon Density values. The "Elderly" cluster is considered a basic theme with a centrality of 0.63 and a density of 32.80. Similarly, "Cardiovascular disease" is also a basic theme but with higher centrality (4.73) and lower density (23.45). "Psychosocial stress" is categorized as an emerging or declining theme, as it has a low centrality of 0.08 and density of 14.29. "Covid-19" is a basic theme with moderate centrality (1.03) and density (22.84). "Psychosocial risk factors" and "Risk factors" are both basic themes with centrality values of 0.28 and 1.69, and density values of 18.57 and 25.92, respectively. The clusters "Mediation analysis," "HIV," "Health inequalities," "Frailty," "Chronic kidney disease," "Frail elderly," "Negative affectivity," "Multimorbidity," and "Self-management" are all categorized as niche themes. These themes have low centrality values ranging from 0.00 to 0.50, indicating their specialization within the research field. The corresponding density values for these niche themes range from 33.33 to 56.56, further emphasizing their focused nature. Themes such as "Deprivation," "Type 2 diabetes," "Quality of life," "Social deprivation," "Cardiac rehabilitation," and "Cardiometabolic risk" are classified as basic themes. They demonstrate moderate centrality values ranging from 0.46 to 0.51 and varying density values between 18.88 and 56.56. Lastly, the "Heart rate variability" cluster stands out as a motor theme with a high centrality value of 0.13 and a density of 56.56. Overall, this summary provides an overview of the different clusters, their classification, and their respective Callon Centrality and Callon Density values, shedding light on the diversity and prominence of research themes within the field.

**Table 2:** Clusters of thematic map.

Clusters	Callon centrality	Callon density	Classification
Elderly	0.63	32.80	Basic
Cardiovascular disease	4.73	23.45	Basic
Psychosocial stress	0.08	14.29	Emerging or declining
Covid-19	1.03	22.84	Basic
Psychosocial risk factors	0.28	18.57	Basic
Risk factors	1.69	25.92	Basic
Mediation analysis	0.00	33.33	Niche
HIV	0.11	38.89	Niche
Health	0.09	33.78	Niche
Deprivation	0.46	18.88	Basic
Type 2 diabetes	0.50	29.17	Basic
Health inequalities	0.00	33.33	Niche
Quality of life	0.15	19.52	Basic
Social deprivation	0.51	23.77	Basic
Frailty	0.00	33.33	Niche
Cardiometabolic risk	0.11	33.33	Niche
Heart rate variability	0.13	56.56	Motor
Cardiac rehabilitation	0.13	25.00	Basic
Chronic kidney disease	0.00	38.89	Niche
Frail elderly	0.00	33.33	Niche
Negative affectivity	0.00	50.00	Niche
Multimorbidity	0.11	33.33	Niche
Self-management	0.00	33.33	Niche

#### Thematic evolution

**Figure 9:**
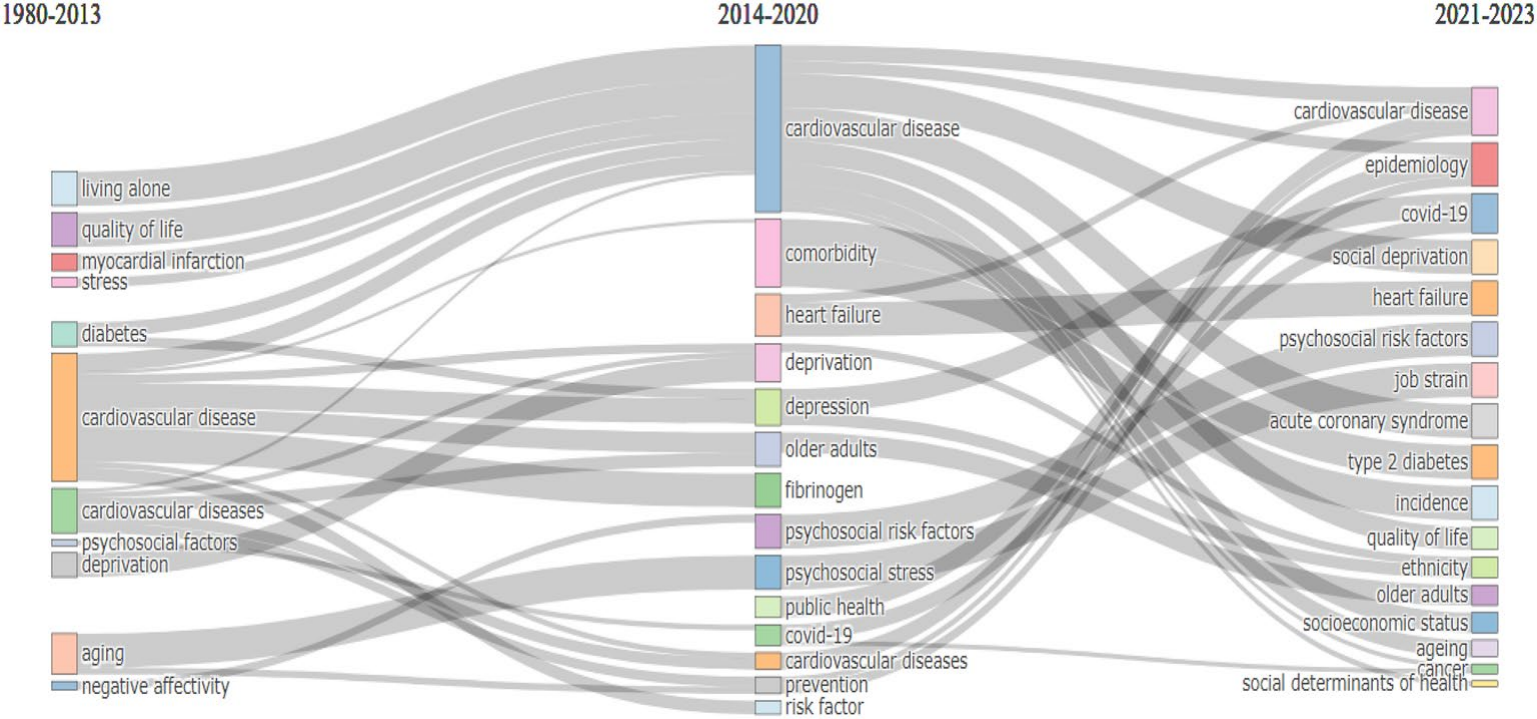
Thematic evolution. 2014 and 2020 were crucial points for the transformation of the main topics. This figure was generated using the Bibliometrix (Version 4.2.2) application and the BibTex data file.

The thematic evolution analysis of the knowledge dynamicity in the field of social isolation and the risk of cardiovascular disease, as depicted in Fig. 9, reveals the transitions in research focus over different time periods. From 1980 to 2013, the prominent themes included "aging," "cardiovascular disease," "cardiovascular diseases," "comorbidity," "depression," "deprivation," "fibrinogen," "older adults," and "risk factor." During this period, the research also encompassed topics like "diabetes," "living alone," "myocardial infarction," "negative affectivity," "psychosocial factors," "quality of life," and "stress." From 2014 to 2020, the research landscape expanded to include emerging themes such as "prevention," "psychosocial stress," and "covid-19." The themes of "cardiovascular disease" and "cardiovascular diseases" continued to be significant during this period, along with "deprivation," "diabetes," "living alone," "myocardial infarction," "negative affectivity," "psychosocial risk factors," and "quality of life." The subsequent period from 2021 to 2023 witnessed further thematic evolution in the field. New themes emerged, including "acute coronary syndrome," "ageing," "cancer," "epidemiology," "social deprivation," "social determinants of health," and "socioeconomic status." Existing themes like "cardiovascular disease," "covid-19," "comorbidity," "heart failure," "older adults," "prevention," "psychosocial risk factors," "job strain," "ethnicity," and "public health" continued to be explored during this time. This analysis highlights the dynamic nature of research in the field of social isolation and the risk of cardiovascular disease, with shifts in focus and the emergence of new themes over time. It provides valuable insights into the evolving knowledge landscape and trends within the field.

#### Trending topics

**Figure 10:**
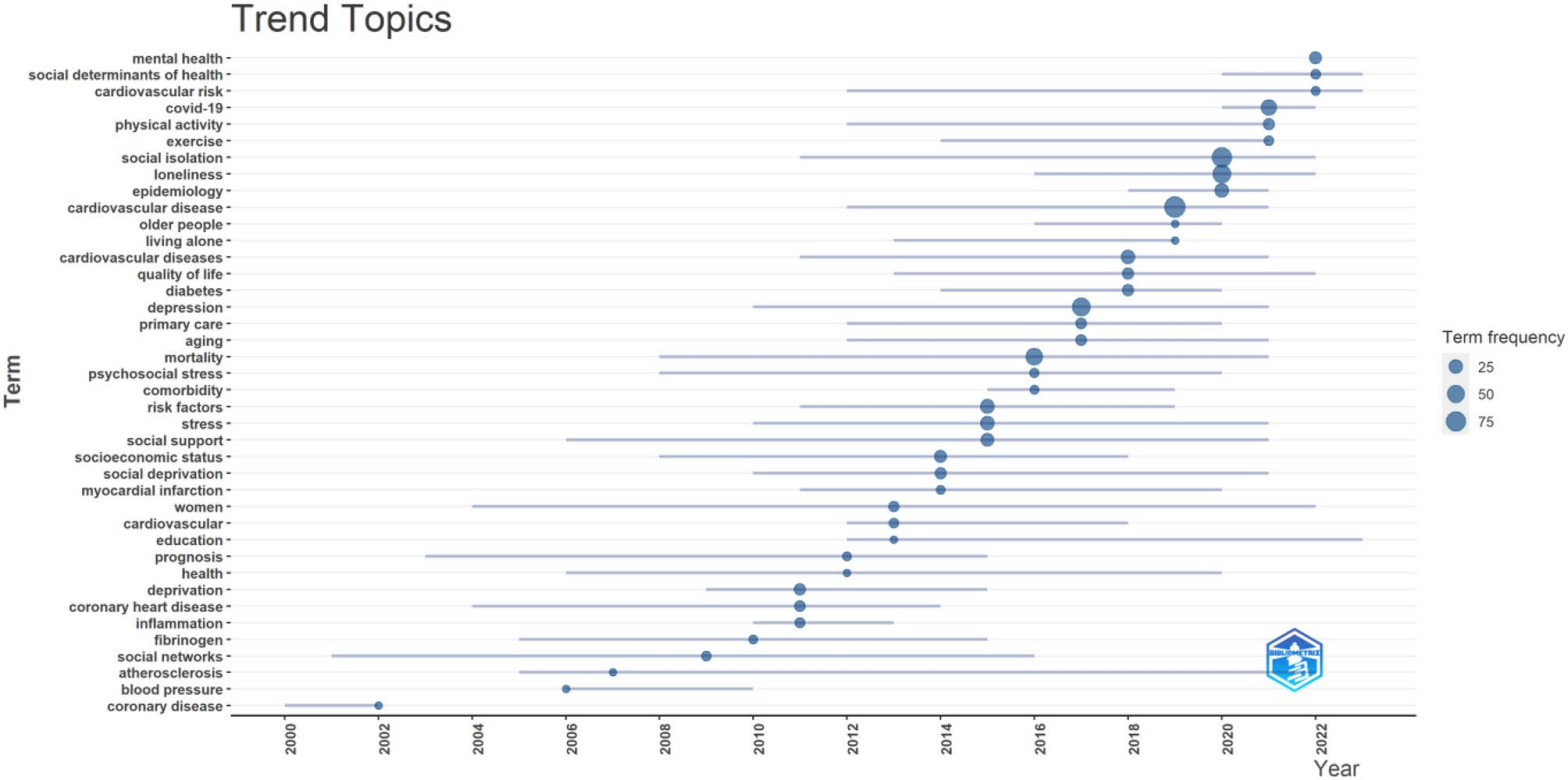
Trending topics. The graph depicts the research topic's time span, with horizontal lines indicating the duration and blue circles representing the frequency of the term. This figure was generated using Bibliometrix (Version 4.2.2) and BibTex data files.

Based on Fig. 10, the most recent trending topics in the field are "mental health," "social determinants of health," and "covid-19." These research areas have gained significant attention and focus in recent times.

## Discussion

The main goal of this research is to improve our comprehension of the relationship between social isolation, loneliness, and risk factors for cardiovascular disease. This will be achieved by conducting a thorough examination of existing literature, combining and analyzing data through statistical synthesis, and performing a bibliometric analysis to gain insights into publication patterns and research trends. Including six studies from different countries, we examined the association between poor social health and incident cardiovascular diseases in different ethnicities. Our review and meta-analysis showed that poor social relationships were associated with a 16% increase in the risk of incident CVD. The strength of the evidence in this review was high, and all studies were adjusted for socio-demographic, behavioral, and psychological factors.

The only previous systematic review that focused on the prospective association between loneliness or social isolation and the first occurrence of CVD reported a 29% increase in the risk of new events ^12^. This review and meta-analysis represent the second one that focuses on the same association. The risk of incident CVD in the current study is comparable to the reported risk in this previous systematic review and may even be higher, as this study included only six studies, whereas the previous review included eleven studies.

The prevalence and risk for social isolation and loneliness appear highest for old and retired people^45,46^. Risk and protective factors including physical health conditions, psychological and cognitive factors for social isolation and loneliness have been well described^11,47,48^. In addition, there is a bidirectional relationship between risk factors and social isolation, or loneliness. Thus, covariates adjusted for these risk factors are very important to establish a significant relationship between social health and the occurrence of incident CVDs. In this study, adjustment for possible risk factors was applied in all studies, and the pooled effect estimate was very significant, indicating that poor social relationships are strongly associated with incident CVD.

The theme of "cardiovascular disease" encompasses a wide range of terms that are relevant to understanding the various aspects of this health condition (Fig. 8). These terms include "social isolation," "depression," "loneliness," "mortality," "cardiovascular diseases," "stress," "social support," "anxiety," "mental health," "socioeconomic status," "older adults," "physical activity," "aging," "coronary heart disease," "women," "aged," "cardiovascular," "exercise," "social determinants of health," "cardiovascular risk," "cohort studies," "health disparities," "lifestyle," "atherosclerosis," "blood pressure," "depressive symptoms," "education," "covid-19 pandemic," "gender," "personality," "psychology," "social environment," "USA," "cohort," "cortisol," "heart disease," "heart rate," "lockdown," "marital status," "marriage," and "women's health." These terms highlight the multidimensional nature of CVD research, covering factors such as social, psychological, and environmental determinants, as well as their interplay with various demographic and health-related variables.

Figure 10 presents the trending topics in the field, focusing on "mental health," "social determinants of health," and "covid-19." These topics have gained significant attention and prominence in recent times. "Mental health" has become a prominent area of research and discourse, indicating a growing recognition of the importance of mental well-being. This topic encompasses various aspects related to mental health, including mental disorders, psychological well-being, mental health interventions, and the impact of mental health on overall health outcomes^49^. "Social determinants of health" refers to the social, economic, and environmental factors that influence health outcomes. This topic explores the relationship between social factors (such as socioeconomic status, education, employment, and social support) and health disparities. Understanding the social determinants of health is crucial for addressing health inequities and developing effective public health interventions^50,51^. "Covid-19" has gained global attention due to the ongoing pandemic caused by the novel coronavirus. Research related to Covid-19 encompasses a wide range of areas, including epidemiology, transmission dynamics, clinical manifestations, prevention and control measures, vaccine development, mental health implications, and the social and economic impact of the pandemic^52,53^. These trending topics reflect the current priorities and concerns within the field, highlighting the significance of mental health, the role of social determinants in shaping health outcomes, and the profound impact of the Covid-19 pandemic on various aspects of health and society.

## Strength and limitations

The strongest point in this study is being longitudinal, as this allowed us to comment on the direction of the relationship between social relationships and CVD and avoid the problem of reverse causation. The second point is the covariate analysis used in all studies in this review. Statistical adjustment for factors that are likely to be on the causal pathway, such as depression or health-related behavior, minimizes the risk of bias. This study has some limitations, one of which is that we only searched four databases and English-language content. Although, they are the most commonly searched databases, still this may subject to selection bias. In addition, we restricted the inclusion to studies that report only hazard ratios. The limitation of bibliometric studies is the reliance on a single database to extract data, which is often restricted to English-language publications.

As this is an observational study, we cannot infer causality from our findings, nor can we exclude confounding by unmeasured common causes or reverse causation if deficiencies in social relationships are the result of subclinical disease. It is unclear which (social isolation or loneliness) matters most for CVD outcome. Most of the studies did not present or explicate a conceptual causal model to guide their selection of variables, including proposed mediators and moderators.

## Conclusions

Poor social health increases the risk of and worsens outcomes in incident cardiovascular diseases. However, the observed effect estimate is small, and this may be attributable to residual confounding from the incomplete measurement of potentially confounding or mediating factors. Our results emphasize the importance of having intervention studies to reduce the impact of poor social health on incident cardiovascular diseases and mortality among older adults. Bibliometric analysis of research on social isolation, loneliness, and CVD revealed hotspots, spatiotemporal positioning, a rich thematic structure, and thematic evolution from 1980 to 2023. Steptoe, A., affiliated with the University College London, United Kingdom, is the most prolific scholar. The United States is the most cited and productive country. Analysis of the thematic evolution of knowledge on social isolation and the risk of cardiovascular disease revealed the transitions in research focus over different time periods. Various emerging themes, including mental health, social determinants of health, and COVID-19, were observed. Overall, this bibliometric study provides valuable insights into the research on social isolation, loneliness, and CVD. This information can be used to inform future research and to develop interventions to reduce social isolation and loneliness, and ultimately to improve the cardiovascular health of the population.

### Supplementary Information

Supplementary Information.

## Data Availability

The datasets generated and/or analyzed during the current study are not publicly available but can be obtained from the corresponding author [OA] upon reasonable request.
