# Supplementary material for: The impact of social isolation and loneliness on cardiovascular disease risk factors: a systematic review, meta-analysis, and bibliometric investigation
Source: Sci Rep. 2024 Jun 4;14:12871. doi: 10.1038/s41598-024-63528-4 (PMC11150510; doi:10.1038/s41598-024-63528-4)
Supplement: Supplementary file 1 — Supplementary Information. [file 41598_2024_63528_MOESM1_ESM.pdf]

Appendix 1: Overview of studies included in the review focusing on the data source, data collection dates, group under the study, age of the subjects at the baseline, follow-up period and the term used under the studies

"First author & Year published"	"Data source & country"	"Data collection dates"	Population group (number of participants)	Age of study subjects at baseline	Follow-up period- mean, median and/or range	Term used in the paper
**Freak- Poli 2021**	Aspirin in Reducing Events in the Elderly (ASP REE) trial/Australia	2010-2014 to 2017	Older adults (n=11,486)	70 years and over	Median 4.5 years	Social isolation, loneliness and social support
**Golaszewski 2022**	Women's Health Initiative Extension Study II/ US	2014- 2015 to 2019	Postmenopausal women in the US (n= 57825)	65 to 99 years	Study length; 4-5 years	Social isolation and loneliness and social support
**Novak 2020**	Swedish national registers in Gothenburg/ Sweden	2000- 2012	Male and female (n = 524)	70 years old	12- year follow -up period	Loneliness
**Christiansen 2020**	2013 Danish "How are you?" survey/Denmark	2013 – 2018	Male and female (n=24,687)	35 – 79 years	5-year follow-up period	Loneliness and social isolation
**Feifei 2020**	Wave 4 participants of English Longitudinal Study of Ageing (ELSA)/ England	2008/9-2017/18.	Older adults (n=58 50)	Over 50 years	9.6 years	Loneliness and social isolation
**Gronewold 2020**	Population-based Heinz Nixdorf Recall study/ Germany	2000- 2003 to 2017	Men and women (n=4139)	45 – 75 years	Median of 13.41<br>3.4 - years	Social support (instrumental, emotional and financial) and social integration

Appendix 2: Overview of studies included in the review focusing on the outcome measures, number of events, additional adjusted models and the main results

"First author & Year published"	Outcome measure	Number of events	Covariates adjustment	Main results
**Freak- Poli 2021**	The main outcomes were incident CVD and fatal CVD,<br>and subtypes are major adverse cardiovascular events (MACE), heart failure hospitalization, MI and stroke.	487 (4.2%) first time CVD events and 83 (0.7%) CVD deaths.	Additional models adjusting for sociodemographic, lifestyle and depressive symptoms	Individuals with poor social health were 42 % more likely to develop CVD (p = 0.01) and twice as likely to die from CVD (p = 0.02) over a median 4.5 years' follow-up.<br>Interaction effects indicated that poorer social health more strongly predicted CVD in smokers (HR 4.83, p = 0.001, p-interaction = 0.01), major city dwellers (HR 1.94, p < 0.001, p-interaction= 0.03), and younger older adults (70-75 years; HR 2.12, p < 0.001, p- interaction = 0.01).<br>Social isolation (HR 1.66, p = 0.04) and low social support (HR 2.05, p = 0.002), but not loneliness (HR 1.4, p = 0.1), predicted incident CVD.<br>All measures of poor social health predicted ischemic stroke (HR 1.73 to3.16).
**Golaszewski 2022**	Major CVD<br>including coronary heart disease, stroke,<br>and death from CVD	1599 major CVD events	Adjusting for age, race and ethnicity, educational level, and depression and relevant health behavior and health status	Continuous scores of social isolation and loneliness and Hazard ratios (HRs) and 95% CIs for CVD; the HR for the association of high vs low social isolation scores with CVD was 1.18 (95% CI, 1.13-1.23), and the HR for the association of high vs low loneliness scores with CVD was 1.14 (95% CI, 1.10- 1.18).<br>The HRs after additional adjustment for health behaviors and health status were 1.08 (95% CI, 1.03-1.12; 8.0% higher risk) for social isolation and 1.05 (95% CI, 1.01- 1.09; 5.0% higher risk) for loneliness.<br>Women with both high social isolation and high loneliness scores had a 13.0 % to 27.0 % higher risk of incident CVD than did women with low social isolation and low loneliness scores.

**Novak 2020**	CVD death, all cause of death.	Cardiovascular disease death accounted for 59.2% (n = 84)	Adverse socioeconomic status, health and health related behavioral factors that have previously been shown to be associated with loneliness.<br>Current perceived economic situation was assessed using a seven- point scale ranging from excellent to very bad (coded from 1 to 7).<br>Previous history of having (yes/no) cancer, diabetes, coronary heart disease and stroke was based on self- report as well as from medical examinations conducted by a study physician.	A total of 142 participants died during the 12- year follow- up period, with 5334 person- years at risk, corresponding to 26.6 deaths/1000 person- years. Cardiovascular disease accounted for 59.2% of all deaths. The cumulative rates/ 1000 person- years for cardiovascular mortality were 20.8 (men) and 11.5 (women), and for all- cause mortality 33.8 (men) and 20.5 (women), respectively. In Cox regression models, no significant increased risk of mortality was seen for men with loneliness compared to men without loneliness (cardiovascular mortality HR 1.52, 95% CI 0.78– 2.96; all- cause HR 1.32, 95% CI 0.77– 2.28).<br>Increased risk of cardiovascular mortality was observed in women with loneliness compared to those without (HR 2.25, 95% CI 1.14– 4.45), and the risk remained significant in a multivariable- adjusted model (HR 2.42, 95% CI 1.04– 5.65).
**Christiansen 2020**	Cardiovascular disease [CVD], chronic obstructive pulmonary disease [COPD], diabetes mellitus Type 2 [T2D], and cancer.	1,651 (6% of the full sample) were diagnose d with CVD.	Additional models adjusting for sociodemographic, behavioral, and psychological factors	Cox proportional hazard regression analyses showed that loneliness and social isolation (SI) were independently associated with CVD (loneliness: adjusted hazard ratio (AHR)= 1.20, 95% confidence interval [CI;1.03,1.40]; SI: AHR = 1.23, 95% CI [1.04,146]) and T2D (loneliness: AHR =1.90, 95% CI [1.42, 2.55]; SI: AHR = 1.59, 95% CI [1.15, 2.21]).
**Feifei 2020**	CVD events and CVD death.	997 (17%) CVD events	Risk index were used to avoid multi-collinearity, taking into account a wide range of modifiable risks factors that were well established in the literature, including obesity, high cholesterol, hypertension, diabetes, smoking, diet,	17% participants reported having newly diagnosed CVD and 16% had a CVD- related hospital admission. Loneliness was associated with an increased risk of CVD events independent of potential confounder s and risk factors. The hazard of people with the highest level of loneliness was about 30% higher for onset CVD diagnosis (HR: 1.05, 95% CI: 1.01 to 1.09) and 48% higher for CVD- related hospital admissions (HR: 1.08, 95% CI: 1.03 to 1.14), compared with the least lonely.

			physical activity, abnormal sleep and depression.	There was little evidence that social isolation was independently associated with the risk of either CVD diagnosis or admission.
**Gronewold 2020**	Incident fatal and non-fatal cardiovascular events and all- cause mortality.	339 cardiovascular events (122 strokes, 183 coronary events, 34 deaths related to diseases of the circulatory system) and 530 deaths (98 cardiovascular, 432 other causes).	Minimally adjusted model (adjusting for age, sex, social integration or social support, respectively); biological model (minimally adjusted +systolic blood pressure , low- density and high-density lipoprotein cholesterol, glycated hemoglobin, body mass index, antihypertensive medication, lipid- lowering medication and antidiabetic medication); health behavior model (minimally adjusted +alcohol consumption, smoking and physical activity); socioeconomic model (minimally adjusted +income, education and employment); and depression model (minimally adjusted +depression, antidepressants and anxiolytics).	Lack of financial support was associated with an increased cardiovascular event risk (minimally adjusted HR=1.30(9 5%, CI1.01 to 1.67)).<br>Lack of social integration (social isolation) was associated with increased mortality (minimally adjusted HR=1.47 (95% CI 1.09 to 1.97)).<br>Effect estimates did not decrease to a relevant extent in any regression model.<br>.

Appendix 3: Measurement and prevalence of loneliness and social isolation in the studies included in our review with special emphasis to the measurement scales, number of events and scoring and categorization.

"First author & Year published"	Term used in the paper	Measurement	Number of items	Scoring and categorization
**Freak- Poli 2021**	Social isolation and loneliness and social support.	Revised Lubben Social Network Scale for social support and Center for Epidemiological Studies – Depression (CESD) Scale for loneliness.	1,5, and 6	For sensitivity analyses, two approaches were employed to calculate social health measures as continuous. First, each response category was sequentially numbered.<br>Second, the response categories were recoded as values, for example, "three-four" became 3.5. In the second scenario, "nine or more" was recoded as 9, "sometimes" as 2, "often" as 8, and "always" as 24.<br>Social isolation scales ranged from 0-21 (scenario 1) and 0- 85 (scenario 2), social support from 0- 20 and 0-36, and loneliness from 0- 3 and 0-6.<br>The social health composite categories were defined using the binary categories of social health as positive (not isolated, supported, and not lonely), or poor (isolated, not supported and/or lonely).
**Golaszewski 2022**	Social isolation and loneliness and social support.	Social isolation index score, UCLA<br>Loneliness Scale and Medical Outcomes Study Social Support Survey.	7- items for social isolation score,<br>3- items for loneliness and<br>9- items for social support	Social isolation; Participants responded "yes" (1) or "no" (0) to the following questions: "Are you currently married or in an intimate relationship with at least 1 person?" and "Do you live alone?"<br>Participants also responded on a 4-point Likert scale ("rarely or never" [1], "once a month" [2], "several

				times a month" [3], or "at least once a week" [4]) to the question "How often, if at all, do you do any of the following activities?<br>(a) Meet with family or friends who do not live with you; (b) Communicate with family or friends by phone or email; (c) Go to a church or other religious center; d) Go to a cultural event such as a movie, concert, play, or lecture; e) Eat out of the house; and (f) Go shopping<br>." Each activity was dichotomized such that a response of "rarely or never" was coded as 1 and all other response s were coded as 0.<br>With use of methods previously implemented among older adults, the social isolation index score (range, 0-8) was calculated as the sum, with higher scores indicating greater social isolation. Loneliness; For example, 1 item used was "How often do you feel you lack companionship?" Response options (on a 3- point Likert scale) were "hardly ever or never" (1), "some of the time" (2), and "often" (3).<br>Scores were summed and divided by 3 to provide a mean loneliness score (1-3), with higher scores indicating greater loneliness.<br>For social support; participants were given the following prompt: "People sometimes look to others for help, friendship, or other types of support.

				Next are some questions about the support that you have. How often is each of the following kinds of support available to you if you need it?" For example, 1 item included "Someone you can count on to listen to you when you need to talk," with response options on a 5-point Likert scale ranging from "none of the time" (1) to "all of the time" (5). A total score (range, 9-45) was calculated, with higher scores reflecting greater social support.
**Novak 2020**	Loneliness	Single question loneliness scale as 'do you feel lonely? '	1- item with four alternative responses	Self- perceived feeling of loneliness was assessed by a single question as 'do you feel lonely?' There were four alternative responses where 1 indicated never feeling lonely, 2 seldom, 3 sometimes, and 4 very often. The four categories were then merged into a dichotomous variable as 0 = not lonely (responses 1–2), and 1 = lonely (responses 3–4). Cardiovascular deaths were those with International Classification of Diseases, 10th Revision (ICD-10) codes I.00-I.99.
**Christiansen 2020**	Loneliness and social isolation.	Loneliness was assessed using the Three- Item Loneliness Scale [T- ILS]. Social isolation was measured using a simple index inspired by the detailed Social Network Index create d by Berkman and Syme.	3- items for loneliness and 5- items for social isolation	The T-ILS contains the following questions: How often do you feel isolated from others? How often do you feel you lack companionship? How often do you feel left out? The sum of the items (ranging from 3 to 9) provides a global measure of loneliness, with higher scores

				indicating greater loneliness. The following five indicators were included in social isolation index: (a) living alone (yes = 1 and no = 0); (b) less than monthly contact with family with whom one does not live (yes = 1and no = 0); (c) less than monthly contact with friends (yes = 1 and no = 0); (d) less than monthly contact with colleagues/ fellow students outside the workplace or school (yes = 1 and no = 0); and (e) less than monthly contact with neighbors or the local community (yes = 1 and no = 0). Using the five indicators, we generated a sum score ranging from 0 to 5, with higher scores indicating greater SI. A score between 3 and 5, corresponding to a maximum of two areas of social interfaces, was treated as an indicator of SI.<br>CVD was defined as ischemic heart diseases (DI20– DI25), heart failure (DI150), peripheral artery occlusive disease (DI170– DI174), and stroke (DI160– DI164).
**Feifei 2020**	Loneliness and social isolation.	Loneliness was measured using the three- item subscale from the revised University of California, Los Angele s loneliness scale. Social isolation was measured using five- item Shankar index.	3- item s for loneliness and<br>5- items for social isolation	The questions for loneliness include:<br>(1) How often do you feel lack companionship?<br>(2) How often do you feel isolated from others?<br>(3) How often do you feel left out?<br>Responses to each question were scored on a 3- point Likert scale ranging from hardly ever/never, to some of the time, to often.

				Using the sum score, we had a loneliness scale ranging from 3 to 9, with a higher score indicating increased loneliness. The distribution of loneliness was positively skewed (skewness=1.25).<br>In social isolation, participants were assigned one point for each of the following seven items: living alone, having less than monthly contact with children, relatives and friends, not belonging to any social organization or club, not working and not volunteering.<br>Different from the study by Shankar et al, we considered only meeting in-person and speaking on the telephone as the questions on writing/emailing had low factor loadings when tested in factor analysis. While the original scale included five items, we added working and volunteering to take into account any social contact through the network of colleagues.<br>The social isolation scale ranged from 0 to 7, with the distribution being slightly skewed (skewness=0.31).
**Gronewold 2020**	Social support(instrumental, emotional and financial) and social integration.	Instrumental and emotional social support were measured with a German adaptation of the New Haven Established Populations for Epidemiologic Studies of the Elderly (New Haven EPESE) questionnaire in a computer- assisted personal interview. For financial support,	11-items related to social and productive activity.	Instrumental and emotional social support first asks for the availability of someone to help in daily tasks like buying groceries, house cleaning, preparing meals (instrumental support) and someone to approach in case of emotional problems (emotional support). If support was

		self- constructed questions analogue to the New Haven EPESE were used. Additionally, the participants could specify if they had refused offered financial support.<br>Social integration with the social integration index developed by Berkman et al.		available, participants were asked who actually provided support during the previous 12 months or whether support was not needed during that time. If participants reported persons who actually provided support, they were asked whether that support had been sufficient. For financial support, self-constructed questions analogue to the New Haven EPESE were used. Additionally, the participants could specify if they had refused offered financial support. Based on the combination of this information, four categories were defined: 'support not needed' (someone available but not needed), 'support adequate' (someone provided support, this support was sufficient), 'support inadequate' (someone provided support, this support was insufficient) and 'no support available' (no one available). Lack of instrumental or emotional support was defined as one of the latter two categories; for financial support, refusal of offered support was additionally classified as lack of support. The index includes three types of ties: (1) marital status/cohabitation, (2) contacts with close friends/family and (3) affiliation with voluntary associations. These types of ties each scored from 0 to 2, thus the index ranged from 0 to 6. Marital status/cohabitation was scored

				as 0 if the participant was single, divorced or widowed and 2 if the subject was married or living with a partner. Contacts with close friends and family was scored as 0 in case of 0–2 contacts, 1 in case of 3–11 contacts and 2 in case of ≥12 contacts. Affiliation with voluntary associations was defined by membership in any of six types of political, religious, community, sports or professional organizations and scored as 0 in case of no membership, 1 in case of membership in one organization, and two in case of membership in ≥2 organizations. The social integration index was categorized into four levels: level I included persons who scored 0 or 1, and levels II, III and IV included persons who scored 2–3, 4–5 and 6, respectively. We defined lack of social integration (social isolation) by level I.

Appendix 4: Measurement and prevalence of loneliness and social isolation in the studies included in our review with special emphasis to the assessment of reliability and validity, how many times the events measured and the effect estimates related to outcome measures.

"First author & Year published"	Prevalence of loneliness/isolation /social support	Comments related to reliability, validity, responsiveness and/or interpretability	How many times were social relationships measured?	Effect estimates related to incident CHD and/or stroke
**Freak- Poli 2021**	Social isolation (2%), low social support (2%) and loneliness (5%).	Revise d Lubben Social Network Scale (LSNS- R) has very good reliability as measured by the internal consistency Cronbach Alpha value of .78. The correlation coefficient between the original LSNS and LSNS-R was 0.68.<br>Center for Epidemiological Studies – Depression (CESD) Scale has high internal consistency (Cronbach's αs .85−.90).	Once, at baseline	Poorer social health more strongly predicted CVD in smokers (HR 4.83, p=0.001, p-interaction = 0.01), major city dweller s (HR 1.94, p<0.001, p- interaction=0.03), and younger older adults (70-75 years; HR 2.12, p<0.001, p- interaction = 0.01).<br>Social isolation (HR 1.66, p=0.04) and low social support (HR 2.05, p=0.002), but not loneliness (HR 1.4, p=0.1), predicted incident CVD.<br>All measures of poor social health predicted ischemic stroke (HR 1.73 to 3.16).
**Golaszewski 2022**	Social isolation; (56.5% low score, 43.5 % high score; scores range from 0-8. High score above the median of 1, below score below the median of 1)<br>Loneliness; 65.3% low score, 34.7% high score; scores range from 1-3. Low score below the median of 0.33, high score above the median of 0.33).<br>Social support score 38.1	A social isolation index score has acceptable internal consistency, with an alpha of .73 and moderate to strong item- rest correlations.<br>Loneliness scale has shown	Once, at baseline	Hazard ratios (HRs) and 95% CIs were estimated from sequentially adjusted Cox proportional hazards regression models as follows: model 1 was adjusted for age, race and ethnicity, educational level, and history of depression; model 2was additionally adjusted for social isolation or loneliness to test for independent associations of each exposure variable; model 3 added health behaviors (smoking, alcohol consumption, and physical activity); and model 4 added health status variables (history of diabetes, hypertension medication use, hyperlipidemia medication use, general health, and physical functioning).<br>Models 2 through 4 were then fit with an interaction term (social isolation × loneliness) to allow more flexibility when estimating the CVD risk ratio comparing

				high social isolation and high loneliness scores vs low social isolation and low loneliness scores.
**Novak 2020**	Loneliness 24.4%	Single- item assessment may result in under reporting due to the stigma associated with being identified as lonely. However, more commonly used and have been shown to predict mortality in elderly.	Once, at baseline	Kaplan Meier analysis showed no significant difference in survival between men with and without loneliness for either cardiovascular- or all- cause mortality (log rank, p > 0.05). For women, Kaplan Meier curves showed no significant difference in all- cause mortality by loneliness status, but a lower survival rate was observed among women with loneliness compared to women with no loneliness regarding cardiovascular mortality (log rank, p = 0.017).no significant increased risk of mortality was observed for men with loneliness compared to men with no loneliness for both cardiovascular (HR 1.52 95% CI 0.78– 2.96) and all- cause mortality (HR 1.32, 95% CI 0.77– 2.28).<br>In all- cause mortality, no significant increased risk of mortality was seen for women with loneliness compared to women with no loneliness (HR 1.64, 95% CI 0.98– 2.76). However, women with loneliness had significantly higher risks of cardiovascular mortality compared to women with no loneliness (HR 2.25, 95% CI 1.14– 4.45). The high risks of cardiovascular mortality in women remained significant in the multivariable- adjusted model (2.42, 95% CI 1.04– 5.65).
**Christiansen 2020**	Loneliness (18%), social isolation (11%) and	The T- ILS has good psychometric properties, including good internal consistency (α =.76 in this sample), a high concurrent, and discriminant validity and correlates strongly	Once, at baseline	Fully adjusted Cox proportional hazard regression analysis<br>Demonstrated that neither loneliness nor SI predicted being diagnosed with CVD, T2D,

	(5%) were both lonely and socially isolated.	with the UCLA loneliness scale. Several larger population- based studies have used a similar scale for social isolation. The sum score was used in sensitivity analyses.		COPD, or cancer when all sociodemographic and explanatory factors were added to the model.
**Feifei 2020**	Loneliness (4.14%) Social isolation (2.33%)	The revised University of California, Los Angeles loneliness scale, is a validated and widely used tool.	Once, at baseline	The effect of social isolation was attenuated after controlling for loneliness and potential confounders. Loneliness (HR: 1.06, 95%CI: 1.02 to 1.11), was a persistent predictor of CVD independent of sociodemographic factors and social isolation (model III). The association between loneliness and CVD held even after controlling for baseline CVD risk (model IV). One-point increase in loneliness was associated with a 5% increase in the hazard of CVD (95%CI: 1.01 to 1.09), meaning that the hazard was 30% higher for people with the highest loneliness score than the lowest.
**Gronewold 2020**	Lack of instrumental support (n=501), Lack of emotional support (n= 659), Lack of financial support (n= 907), and Lack of social integration (n=309).	New Haven EPESE have greater validity among diverse group of older men and women.<br>Berkman- Syme Social Network Index allows researchers to categorize individuals into four levels of social connection, its validated and commonly used for social integration measurement.	Once, at baseline	Sex- stratified and age- stratified analyses showed that; the association between lack of financial support and increased cardiovascular event incidence was stronger for men (minimally adjusted HR 1.54 (95% CI 1.14 to 2.07)) than for women (0.89 (95% CI 0.55 to 1.42)) and stronger for younger (< 65 years, 1.53 (95% CI 1.08 to 2.16)) than older (≥ 65 years, 1.12 (95% CI 0.78 to 1.60),) participants.<br>The association between lack of social integration and cardiovascular events was also stronger for men (1.92 (95% CI 1.07 to 3.46)) than for women (1.12 (95% CI 0.66 to 1.90) and stronger for younger (<65 years, 1.79 (95% CI 1.01 to 3.18)) than older (≥65 years, 1.10 (95% CI 0.64 to 1.91) participants.

				Similarly, the association between lack of social integration and all- cause mortality was also stronger for men (2.45 (95% CI 1.54 to 3.87)) than for women (1.11 (95% CI 0.77 to 1.62) and stronger in younger (1.87 (95% CI 1.22 to 2.88)) than in older (1.17 (95% CI 0.78 to 1.74) participants.

Appendix 5: Criteria for assessment of the risk of bias

	Domain	Lower risk	Higher risk	Unclear risk
**1**	Selection bias	Random allocation	No randomization	No information available
**2**	Detection bias	Assessors were blinded to exposure.	Assessors were not blinded to exposure.	No information available
**3**	Bias due to missing data (Differential loss to follow-up)	Subjects lost to follow-up did not significantly differ from the rest of the sample.	There were significant differences in characteristics likely to increase risk of bias between the baseline data reported for the whole sample and the baseline data of subjects lost to follow-up.	No information available
**4**	Bias due to exposure measurement error	Available data suggest that the tool used to measure loneliness and/or social isolation was comparatively valid and reliable.	Loneliness and/or social isolation were assessed using a tool that was of limited validity and/or reliability.	No information available
**5**	Bias due to outcome measurement error	Measure based on information from medical records, registers and/or death certificates.	Reliance on self-report of diagnosis.	No information available
**6**	Selective reporting (reporting bias)	All outcomes were reported	Not all outcomes were reported	No information available
**7**	Confounding bias	Studies controlled for CVD risk factors correlated with loneliness/social isolation, i.e. gender (in mixed samples), age, socio-economic status. Note that measures relating to health (e.g. diabetes, health- behaviors) are not included in this list because of them potentially being on the causal pathway.	Studies did not control for age, gender and socioeconomic status.	No information available
